# A Luminex Assay Detects Amyloid β Oligomers in Alzheimer’s Disease Cerebrospinal Fluid

**DOI:** 10.1371/journal.pone.0067898

**Published:** 2013-07-02

**Authors:** Adrianna Z. Herskovits, Joseph J. Locascio, Elaine R. Peskind, Ge Li, Bradley T. Hyman

**Affiliations:** 1 Department of Pathology, Brigham and Women's Hospital, Boston, Massachusetts, United States of America; 2 Massachusetts General Hospital, Memory and Movement Disorders Units Alzheimer’s Disease Research Center, Boston, Massachusetts, United States of America; 3 Department of Psychiatry and Behavioral Sciences, School of Medicine, University of Washington, Seattle, Washington, United States of America; 4 Mental Illness Research and Education Clinical Center, Veterans Affairs Puget Sound Health Care System, Seattle, Washington, United States of America; 5 Department of Neurology, Massachusetts General Hospital, Mass General Institute for Neurodegenerative Disease, Charlestown, Massachusetts, United States of America; Thomas Jefferson University, United States of America

## Abstract

Amyloid beta (aβ) protein assembles into larger protein aggregates during the pathogenesis of Alzheimer’s disease (AD) and there is increasing evidence that soluble aβ oligomers are a critical pathologic species. Diagnostic evaluations rely on the measurement of increased tau and decreased aβ42 in the cerebrospinal fluid (CSF) from AD patients and evidence for oligomeric aβ in patient CSF is conflicting. In this study, we have adapted a monoclonal single antibody sandwich ELISA assay to a Luminex platform and found that this assay can detect oligomerized aβ42 and sAPPα fragments. We evaluated oligomeric aβ reactivity in 20 patients with AD relative to 19 age matched controls and compared these values with a commercially available Alzbio3 kit that detects tau, phosphorylated tau and aβ42 on the same diagnostic platform. We found that CSF samples of patients with AD had elevated aβ oligomers compared to control subjects (p < 0.05) and the ratio of aβ oligomers to aβ42 was also significantly elevated (p < 0.0001). Further research to develop high sensitivity analytical platforms and rigorous methods of developing stable assay standards will be needed before the analysis of oligomeric aβ becomes a routine diagnostic assay for the evaluation of late onset AD patients.

## Introduction

Alzheimer’s disease (AD) is the most common dementia in the elderly, affecting nearly half of people in the United States over the age of 85. Pathological hallmarks that are important for the neuropathologic diagnosis of AD include the assessment of amyloid beta (aβ) deposits, neurofibrillary tangles and neuritic plaques [1,2]. Due to its close proximity with the brain parenchyma, cerebrospinal fluid (CSF) is an excellent surrogate for measuring biochemical and neuropathologic changes in brain tissue that occur during AD progression [3].

The amyloid precursor protein is sequentially processed by β and γ-secretase complexes resulting in two major aβ species terminating at positions Val40 (aβ40) or Ala42 (aβ42). The aβ42 protein has two additional hydrophobic amino acids on its C terminus and is more prone to aggregation than aβ40 peptide [4]. It is believed to play a role in the initial seeding of aβ deposits, and is also a major component of the mature senile plaque [5–7]. Monomeric aβ42 aggregates to form higher order structures including oligomers, protofibrils, and insoluble amyloid fibrils. Many studies have found that the soluble aβ aggregates are toxic, can affect synaptic plasticity and disrupt memory formation [8–13]. However, there is no current consensus regarding the structural composition of the toxic aβ oligomer [14], and current data are inconsistent on whether aβ oligomers are increased in biological fluids during AD pathogenesis [4,15–19].

Measurements of aβ42, total tau and phosphorylated tau can be used to diagnose AD with sensitivity and specificities ranging from 81% to 90% when all three analytes are measured [20–22]. The Alzbio3 assay is a multiparametric assay run on the Luminex platform that simultaneously measures both aβ and tau proteins in the same sample and has been shown to have similar diagnostic accuracy as traditional flat plate ELISAs [23–26]. Luminex assays have the advantage of utilizing less sample volume relative to running replicates of each analyte individually and offer a more efficient workflow with higher sample throughput [27]. The Alzbio3 is a core assay used by the Alzheimer’s Disease Neuroimaging Initiative, a national consortium to validate the use of biomarkers and imaging methods [28] and our laboratory participates in an external quality control program for cerebrospinal fluid biomarkers for this assay [29]. We tested several antibodies previously published to detect oligomeric aβ [4,30,31] and adapted the assay with strongest reactivity against synthetic oligomer preparations to the Luminex platform to facilitate comparison of these analytes in CSF.

Previous studies have used a variety of reagents and techniques to assess the presence of aβ oligomers in CSF from AD patients. One study using a Ban50 single antibody sandwich ELISA to detect HMW oligomers documented an elevation of aβ oligomers in CSF that is associated with cognitive decline [4]. Another research group has developed an assay combining flow cytometry with fluorescence resonance energy transfer and found that oligomeric aβ is detectable in non-demented control patients [17] and elevated oligomeric aβ is present in AD cases, although the differences between the groups were not statistically significant [15]. A third strategy utilizing aβ antibody NAB 228 to create an ELISA that detects LMW oligomers did not find any difference between AD and control CSF in their patient population [16]. A fourth approach using the N-terminal aβ antibody HJ3.4 on an Erenna platform, a single molecule fluorescent immunoassay, did not identify oligomeric species in patient CSF [18]. Another recent paper using 3D6 or Nab61 antibodies on the Meso Scale Discovery Platform also did not detect evidence of oligomeric aβ in the CSF [19]. In this study, we developed an oligomeric assay on the Luminex platform and compared our results with the commercially available Alzbio3 assay. We also examined whether there was any correlation between oligomeric aβ and cognitive performance by using Mini Mental State Exam (MMSE), Clinical Dementia Rating (CDR), Immediate and Delayed Recall test scores.

## Materials and Methods

### Subjects

Study participants were recruited from the Clinical Core of the University of Washington (UW) Alzheimer’s Disease Research Center (ADRC). All participants underwent standard neuropsychological evaluation, including Mini-Mental State Examination [32], Clinical Dementia Rating [33], paragraph recalls (immediate and delayed) that were modeled after the Logical Memory subtests I and II of the WMS-R [34] and clinical examination. Informants were also interviewed and clinical diagnosis was assigned at consensus diagnostic meetings. All controls had MMSE scores of ≥ 26, and neither subjects nor informants reported changes in social/occupational functioning suggesting a decline in cognitive function. Diagnosis of probable AD was made based on standard criteria of NINCDS-ADRDA [35]. Following written informed consent, lumbar punctures were performed between 9am and 12:30pm after overnight fasting and the CSF was immediately aliquoted into polypropylene storage tubes and placed on dry ice. Specimens were transferred to a -70 freezer within 15 minutes. Cases were selected by matching the cohorts for age and gender as indicated in Table 1. Subjects were recruited for lumbar punctures at University of Washington and assays were performed at Massachusetts General Hospital.

**Table 1:** Demographic data.

**Demographic**				**AD**	**Control**	**Statistical testing**
		Number of Cases		20	19	
		Gender				
			Male, n	10	9	p > 0.05, ns (Fisher's exact test, p = 1.0, two-sided)
			Female, n	10	10	
		Average age of onset, mean (SD)		72 (5.5)		
		Average age at lumbar puncture, mean (SD)		76 (4.9)	75 (5.4)	p > 0.05, ns (Mann-Whitney U = 173, p = 0.64, two-tailed)
		Years of education, mean (SD)		16 (3.0)	15 (3.0)	p > 0.05, ns (Mann Whitney U = 170, p = 0.57, two-tailed)
**Cognitive Status**						
	Mini Mental Status Exam (MMSE), mean (SD)			21 (5.5)	29 (1.2)	p < 0.05 (Mann Whitney U = 9.0, p < 0.0001, two-tailed)
			Median (Range)	23 (11-28)	29 (26-30)	
	Clinical Dementia Rating Scale (CDRS), mean (SD)			0.9 (0.6)	0.1 (0.2)	p < 0.05 (Mann Whitney U = 10.0, p <0.0001, two-tailed)
			Median (Range)	0.75 (0.5-3)	0 (0-0.5)	
	Immediate Recall Score (IMMSCORE), mean (SD)			3.5 (3.1)	14 (4.3)	p < 0.05 (Mann Whitney U = 13.0, p < 0.0001, two-tailed)
			Median (Range)	2.5 (0-10)	14 (7-21)	
	Delayed Recall Score (DELSCORE), mean (SD)			2 (2.3)	13 (5.2)	p < 0.05 (Mann Whitney U = 8.5, p < 0.0001, two-tailed)
			Median (Range)	0.5 (0-8)	13 (3-21)	
**Genetic Data**						
	ApoE genotype					
			ApoE ε4 carrier, n (%)	16 (80%)	2 (10.5%)	p < 0.05 (Fisher's exact test, p <0.0001, two-sided)
			ApoE ε4 allele frequency	0.475	0.05	

ns = not significant

### Ethics statement

The study protocols were approved by the Institutional Review Board at the University of Washington. Subjects were recruited from University of Washington Alzheimer’s Disease Research Center and written informed consent was obtained from all participants at enrollment. Because AD patients belong to a potentially vulnerable group, a health care surrogate signed the consent forms in addition to the patient (unless the patient was not capable of doing so). Subjects also demonstrated assent by cooperating with and not objecting to any procedures. Participation in this research was strictly voluntary and potential subjects who did not take part in this project were not disadvantaged in any way. Participation in this research project had no impact on eligibility for clinical benefits or services. All aspects of this investigation were conducted according to the principles described in the Declaration of Helsinki.

### Preparation of oligomeric aβ

Oligomers used for assay standards and controls were prepared from monomeric aβ42 according to previously described protocols [4]. Synthetic aβ42 (Peptides International, Louisville, Kentucky) was dissolved in hexafluoro-2-propanol (HFIP) and aliquoted into low protein binding 1.5mL tubes (Thermo, Fisher Scientific; Waltham, Massachusetts) using low retention pipette tips. The HFIP was evaporated and aliquots were stored in a -80 freezer. The aβ42 was dissolved in dimethyl sulfoxide (DMSO) to 1 mM, and then diluted in phenol red free F12 culture medium (US Biological; Swampscott, Massachusetts) to a concentration of 10 µM. The solution was incubated overnight at 4°C to form oligomeric preparations.

### Size Exclusion Chromatography

Size exclusion chromatography was performed on aβ oligomer preparations using an AKTA purifier 10 with two Superdex 75 10/300 GL columns connected in tandem (GE Healthcare, Pittsburgh, PA). Molecular weight calibration was performed using gel filtration calibration standards (GE Healthcare, Pittsburgh, PA) at a flow rate of 1 mL/min. After collection, the fractioned samples were promptly subjected to Alzbio3 or aβ oligomer assay and positive fractions were pooled, aliquoted and stored in a -80°C freezer.

### Preparation of the Oligomeric Luminex, Assay Microspheres

Conjugated Magplex microspheres (Luminex, Austin, Texas) were prepared according to the directions of the manufacturer. In brief, stock microspheres were resuspended in 100 microliters of dH_2_O and transferred to a solution containing 100mM monobasic sodium phosphate, pH 6.2. The magnetic beads were activated by adding 10 microliters of 50 mg/mL Sulfo-NHS followed by 10 microliters of 50 mg/mL EDC (diluted in dH _2_0) and mixed gently by vortex. The microspheres were incubated for 20 minutes in the dark at room temperature with vortexing at 10 minute intervals. The microspheres were washed twice in 250 microliters of 50 mM MES, pH 5.0 with vortex and sonication and separated into three fractions with 5, 25 or 125 micrograms of Ban50 antibody (Takeda Pharmaceutical Company, Osaka, Japan) to optimize binding conditions. In order to couple the capture antibodies to the microspheres, antibodies were incubated in 500 microliters of 50mM MES, pH 5.0 overnight at 4 degrees on a nutator. The following day the antibody-coupled microspheres were washed in PBS –TBN solution (PBS, 0.1% BSA, 0.02% Tween-20, 0.05% Azide, pH 7.4) solution and incubated for 30 minutes at room temperature on a nutator. Two additional washes were performed in 1 mL of PBS-TBN and Ban50-coupled microsphere yield was determined manually by counting on a hemocytometer. To prepare the detection antibody, Ban50-Fab was biotinylated using EZ-Link Sulfo-NHS-Biotin (Pierce Biotechnology, Rockford, Illinois) according to the manufacturer’s directions.

### Measurements of tau, phosphorylated tau and aβ 1-42 in CSF

CSF samples were thawed on ice and reagents from the Inno-Bia Alzbio3 kit (Innogenetics, Gent, Belgium) were allowed to come to room temperature. Microspheres were vortexed, sonicated and diluted in the buffer provided by the kit manufacturer. The conjugate 1 working solution was prepared by diluting the provided biotinylated secondary antibodies. After prewashing the plate, 100 microliters of diluted bead mix were transferred to each well. The plate was aspirated using a vacuum manifold. All measurements were performed in duplicate with 25 µL of conjugate 1 and 75 µL of standards or samples. The plate was incubated overnight on a shaker at room temperature and protected from light.

The following day, detection conjugate solution was prepared with the provided diluent. The plate was washed three times and incubated with detection conjugate for 1 hour at room temperature on an orbital shaker. The plate was washed an additional three times and reading solution was added to each well. The plate was incubated on the shaker for several minutes and read on a FlexMap3D platform (Luminex Corporation, Austin, Texas).

### Measurement of oligomeric aβ in CSF

CSF samples and assay controls were thawed on ice and reagents were allowed to come to room temperature. Ban50 conjugates prepared at 125 micrograms of antibody per 1.25 x 10^6^ microspheres were vortexed and sonicated. A master mix containing Ban50 conjugated microspheres, biotinylated Ban50 detect conjugate and 1% BSA in PBS with protease and phosphatase inhibitors (Roche Applied Science, Penzberg, Germany) was prepared and 25 microliters per well were dispensed into a low retention 96 well black plate assay plate (Corning, Lowell, Massachusetts). Samples and controls were added at 75 microliters per assay well. The plate was incubated overnight on a shaker at room temperature and protected from light with aluminum microplate sealing film.

The following day, the plate was washed three times with PBS, 0.05% Tween and incubated at room temperature with 0.5 µg/mL of Lumigrade Streptavidin-R-Phycoerythrin (Roche Applied Science, Penzberg, Germany) in PBS with 1% BSA for one hour on an orbital shaker. The plate was washed three times and 100 microliters of PBS was added to each well. The plate was incubated on the shaker for several minutes to resuspend the microspheres and read on a FlexMap3D platform (Luminex Corporation, Austin, Texas). Assays were performed on de-identified coded samples. The investigator was blinded to diagnosis, genotype and demographic information during all of the manual steps of assay, and diagnostic groups were compared after the samples were read on the Luminex machine. Ban50 SAS oligomeric assays for AD and control samples were run on the same plate at the same time to minimize variability between assay runs and CSF was analyzed in duplicate wells.

### Cross-reactivity of BAN50 SAS assay with aggregated APP proteolytic fragments

Recombinant sAPPα and sAPPβ proteins (Sigma-Aldrich, St. Louis, Missouri) were oligomerized by incubating one microgram of protein in one milliliter of phenol red free F12 culture medium (US Biological; Swampscott, Massachusetts) at 4°C overnight. Monomeric sAPPα and sAPPβ were prepared by dissolving and evaporating 1 microgram of recombinant protein in HFIP three times before resuspending the protein in 1 milliliter of phenol red free F12 culture medium. Serial dilutions were made in PBS with 1% BSA and protease inhibitors (Roche Applied Science, Penzberg, Germany) starting at 500 nanograms per milliliter. *In vitro* analysis of whether sAPPα interferes with aβ oligomer detection was performed at a range of sAPPα concentrations in the presence of 2% aβ oligomers from pooled SEC fractions. Immunodepletion experiments were performed by preclearing three pools of CSF from different AD patients with Protein A Sepharose (Amersham Pharmacia/GE Healthcare, Pittsburgh, Pennsylvania) for one hour followed by an overnight incubation of the unbound CSF with 2.5 micrograms of 2B3 (IBL International; Ontario, Canada) or IgG control antibody (EMD Millipore, Billerica, MA) on a nutator at 4 degrees. Immunoprecipitation was performed with Protein A Sepharose and unbound CSF was analyzed using the Ban50 assay and read on a FlexMap3D platform (Luminex Corporation, Austin, Texas).

### Statistical analysis

Continuous variables such as tau, phosphorylated tau, aβ42, oligomeric aβ, age at lumbar puncture, years of education, or cognitive testing scores were compared between AD and control groups using two-tailed Mann-Whitney tests or t-tests. Categorical variables, such as gender or ApoE genotype, were compared between AD and control groups using Fisher’s Exact Test. Receiver Operating Characteristic analysis was performed to evaluate test performance and determine appropriate cutoff values for oligomeric aβ assays. Linear regression and Pearson correlations were performed to examine the relation of cognitive tests (MMSE, CDR, Immediate and Delayed Recall) to oligomeric aβ. In experiments performed to assess crossreactivity of the BAN50 assay with sAPPα and sAPPβ, mixed between and within subjects ANOVA (separate biological samples were here treated statistically as “subjects”) was performed relating signal to monomeric and oligomeric sAPP fragments as well as to concentration. Significant main and interaction effects were followed up with post hoc simple main effects and polynomial contrasts across concentration levels. A Greenhouse-Geiser (GG) adjustment to the degrees of freedom (df) was used to adjust for correlated error in tests with df greater than one. In experiments evaluating the detection of oligomeric aβ in the presence of free soluble sAPPα at a range of physiologically relevant concentrations, a one-way repeated measures ANOVA was performed to assess the relation of concentration to signal. Immunodepletion experiments were analyzed using a t-test and confirmed using two nonparametric significance tests. Statistics and graphics were performed using SAS statistical software (Version 9.3), GraphPad Prism (Version 5.0), Origin (Version 8.0), and JMP Pro (Version 10) software.

## Results

### Demographics and Summary Data

An age and gender matched cohort of patients seen at the University of Washington Alzheimer's Disease Research Center between 2004 and 2010 with a diagnosis of AD (n=20) or non-demented control (n=19) were assessed in this study. Demographic factors such as age, gender and years of formal education were not significantly different between the AD and control patient cohorts (Table 1). APOE ε4 genotype was assessed and we observed an overrepresentation of patients with the APOE ε4 allele in the AD cohort (Fisher’s exact test, p <0.0001, two-sided), which is consistent with previous studies finding higher allele frequencies in late onset AD cases [36]. As expected, AD patients performed more poorly on MMSE, Immediate Recall Score and Delayed Recall Score (Mann-Whitney, p < 0.0001, two-tailed). The demographic and clinical characteristics of AD and control cohorts as well as statistical testing comparing both groups are detailed in Table 1.

### Measurements of tau, phosphorylated tau and aβ42

**Figure 1:**
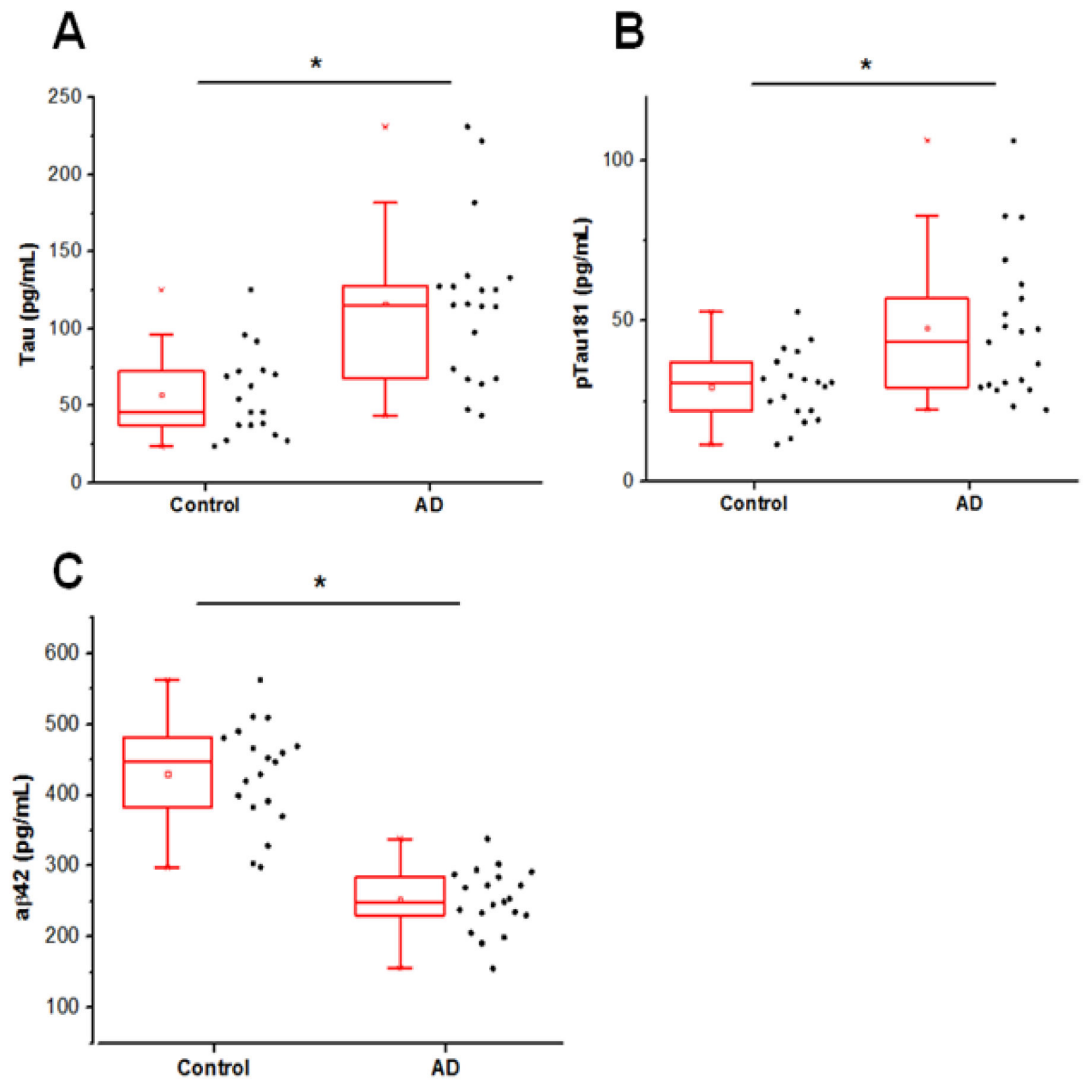
The multiplex AlzBio3 assay is run as a gold standard to distinguish AD and normal cases. **A**. Total tau levels are detected using AT120 and HT7 antibodies (Mann Whitney U = 50, p = 0.0002, two-tailed). **B**. Tau phosphorylated at T181 is detected using a combination of AT270 and HT7 antibodies (Mann Whitney U = 97.5, p < 0.01, two-tailed) **C**. aβ42 is detected using a combination of 4D7A3 and 3D6 antibodies (Mann Whitney U = 4.0, p < 0.0001, two-tailed) * indicates difference between groups is statistically significant with p < 0.05.

A recent study has found that clinical diagnosis is 70.9% to 87.3% sensitive and 44.3% to 70.8% specific for AD, based on a large multicenter comparison of clinical assessments and neuropathologic autopsy data from the National Institute on Aging Alzheimer Disease Centers [37]. The AD and control cohorts were assigned using clinical criteria [35]; we wanted to first examine the expected biomarker profiles of the individuals in these diagnostic groups. As shown in Figure 1, the biomarker profile using the Alzbio3 assay was consistent with prior studies showing decreased aβ42 (Mann Whitney U = 4.0, p < 0.0001, two-tailed), elevated levels of total tau (Mann Whitney U = 50, p = 0.0002, two-tailed) and increased phosphorylated (pT181) tau in the CSF of AD patients (Mann Whitney U = 97.5, p < 0.01, two-tailed) [28]. Baseline studies of CSF from Alzheimer’s Disease Neuroimaging Initiative patients indicate that expected values for tau are 122 (± 58) pg/mL in AD patients and 70 (± 30) pg/mL in cognitively normal control patients; mean values for aβ42 are 144 (± 41) pg/mL in AD patients and 206 (± 55) pg/mL in control patients; and anticipated values for phosphorylated tau at the 181 site are 42 (± 20) pg/mL in AD patients and 25 (± 15) pg/mL in CSF from control patients [38]. Although total coefficients of variation for this CSF analysis are relatively high, ranging from 13 to 36 percent between centers, multiple studies have confirmed that these analytes are robust biomarkers that correlate with AD pathogenesis in clinically defined and autopsy confirmed cohorts [26,38]. The Alzbio3 assays were run on the same plate at the same time to minimize variability between assay runs. In addition to confirming clinical diagnosis, the biomarker profile using the Alzbio3 assay confirms that the protein integrity of the CSF samples should be adequate for detecting oligomers despite the two to eight year sample storage period.

### Oligomeric aβ assay

**Figure 2:**
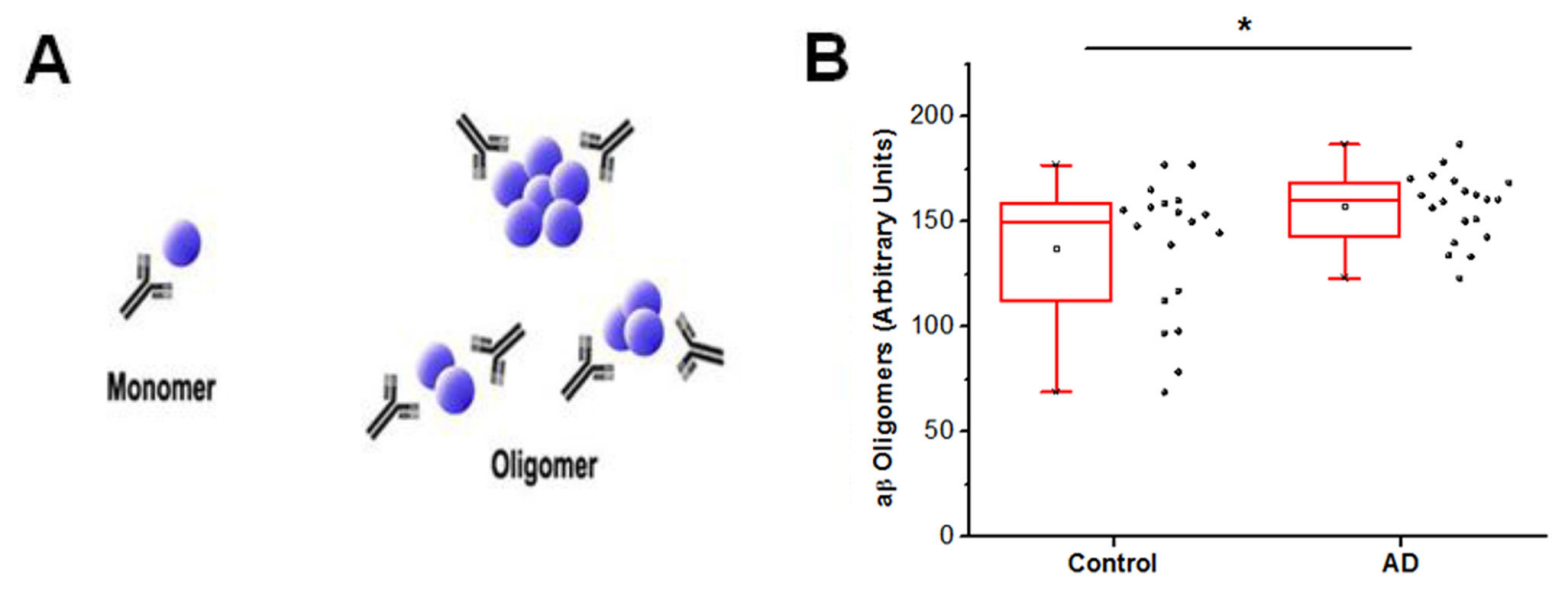
Single antibody sandwich (SAS) assay for oligomeric aβ42 detection in CSF samples. **A**. Schematic of SAS design illustrating the use of the same monoclonal antibody for both capture and detection with aβ42 represented by blue spheres. Monomeric species are not able to bind both capture and detect antibody, however higher order aggregates will have two or more epitopes for Ban50 binding and signal generation. **B**. Using the SAS design with Ban50 antibody, AD cases have more oligomeric aβ42 relative to age and gender matched control CSF (Mann Whitney U = 114, p = 0.03, two-tailed). * indicates difference between groups is statistically significant with p < 0.05.

In order to develop an oligomeric assay for the Luminex platform, we compared the reactivity of two different assay designs using conformational antibodies previously shown to recognize aβ oligomers [4,30,31] or monoclonal single antibody sandwich (SAS) designs [4,16]. Because both conformational and SAS assays should be able to detect oligomers, empirical testing was performed by running several antibody combinations on synthetic preparations of aβ oligomers, selecting the two best assays for a pilot run with patient samples, and performing complete analysis of oligomeric aβ on all biologic samples using the Ban50 SAS assay (Figure 2). We found that the Ban50 SAS assay demonstrated increased signal in AD cases relative to normal controls (Mann Whitney U =114, p = 0.03, two-tailed) and the ratio of oligomeric aβ / total aβ42 was also significantly elevated in AD cases relative to normal controls (Mann Whitney U = 6.0, p < 0.0001, two-tailed).

**Figure 3:**
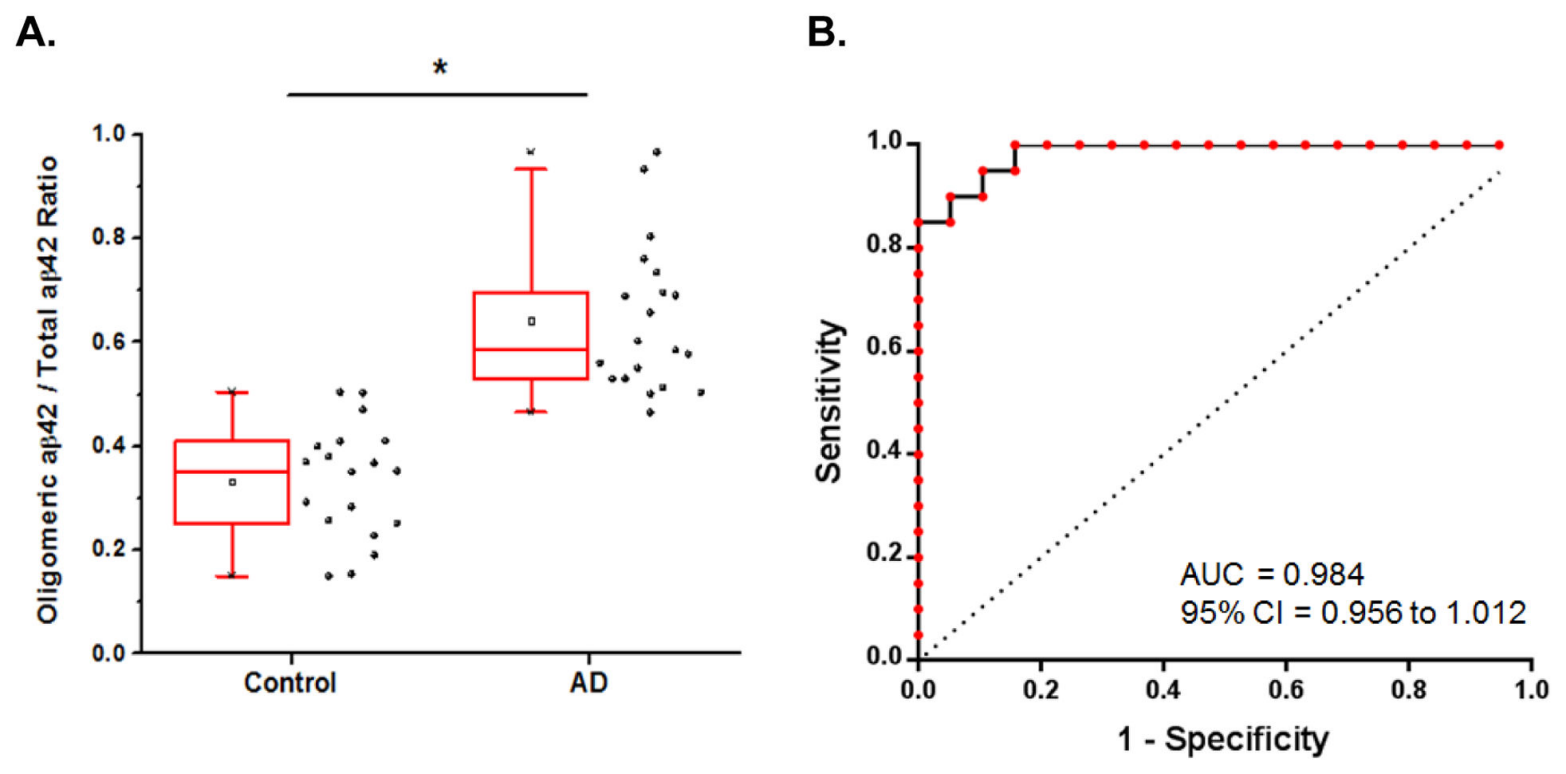
The ratio of oligomeric aβ / aβ42 is a sensitive measure for distinguishing AD and normal cases. **A**. The ratio of oligomeric aβ / aβ42 shows a statistically significant difference between AD and control groups (Mann Whitney U = 6.0, p < 0.0001, two-tailed). * indicates difference between groups is statistically significant with p < 0.0001 **B**. Receiver Operating Characteristic analysis to evaluate test performance yields an Area Under the Curve (AUC) of 0.984 (95 percent confidence interval, 0.956 to 1.012). A cutoff value of 0.503 corresponds to a sensitivity of 90 percent and a specificity of 94.7 percent using the oligomeric aβ / aβ42 ratio.

We performed Receiver Operating Characteristic (ROC) analysis and found that the ratio of aβ oligomers/total aβ42 yields an Area Under the Curve (AUC) of 0.984 (95 percent confidence interval, 0.956 to 1.012). A cutoff value of 0.503 corresponds to a sensitivity of 90 percent and a specificity of 94.7 percent using the oligomeric aβ / aβ42 ratio (Figure 3). Although the ROC analysis indicates that the aβ oligomers/ total aβ42 ratio has excellent performance characteristics, this effect is largely driven by aβ42, raising questions about the added value of measuring oligomeric aβ from a clinical diagnostics standpoint. Because many studies indicate that oligomeric aβ is a major disease relevant toxic species [8–13] and there are a large number of recent clinical trials focused on amyloid clearance, we feel that developing a method to quantify oligomeric aβ may be useful for detecting pathogenic changes during AD progression.

### Correlation between aβ oligomers and cognitive status

**Figure 4:**
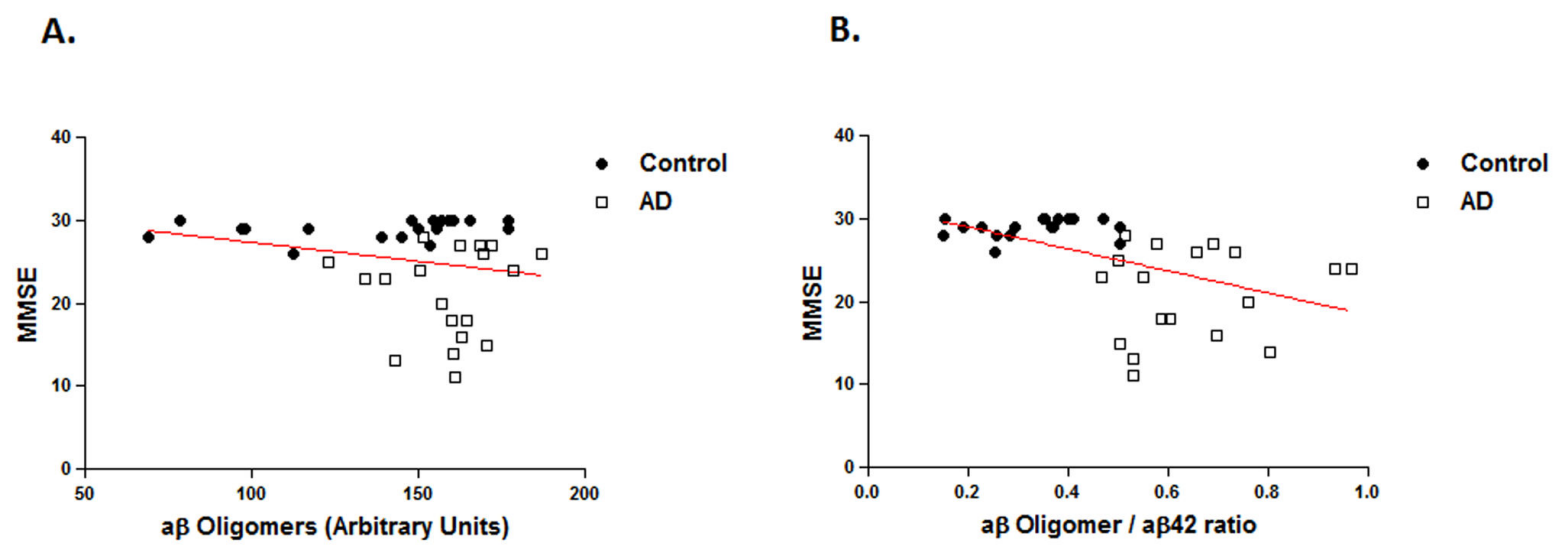
**A**. Correlation with Mini Mental State Examination (MMSE) A. Scatterplot of MMSE and aβ oligomers for the entire sample pooled. The regression line for all samples shows an inverse correlation that is not statistically significant (Pearson correlation, r = -0.225, p = 0.175, n = 38, two-tailed) and is depicted with a solid red line. **B**. Scatterplot of MMSE and the aβ oligomer ratio for the entire sample pooled (solid red line) shows an inverse correlation that is statistically significant (Pearson correlation, r = -0.490, p < 0.005, n = 38, two-tailed).

We also evaluated whether there was any correlation between MMSE and aβ oligomer levels. We observed a modest, not statistically significant negative correlation (Pearson correlation, r = -0.225, p = 0.175, n = 38, two-tailed) between these parameters in the entire sample pooled (Figure 4), and the correlation was also not significant when computed within each diagnosis group separately. We also did not observe a correlation between other cognitive testing parameters such as Immediate or Delayed Recall Scores and aβ oligomer levels (Figure S1). The MMSE and the ratio of oligomeric aβ to aβ42 was negatively correlated for the entire sample pooled (Pearson correlation, r = -0.490, p < 0.005, n = 38, two-tailed), however the correlation was not statistically significant when computed for AD or control cases separately.

### The effect of storage time on assay outcomes

We also examined whether there was any correlation between storage interval and the duration of time that samples were stored for any of the analytes measured in this study (Figure S2). We did not find a statistically significant correlation between storage interval and aβ oligomers (Pearson correlation, r = 0.114, p = 0.49, n=39, two-tailed), aβ42 (Pearson correlation, r = -0.143, p = 0.38, n=39, two-tailed), tau (Pearson correlation, r = -.00065, p = 1.00, n = 38, two tailed), or phosphorylated tau (Pearson correlation, r = 0.014, p = 0.93, n = 39, two-tailed).

### Reactivity of BAN50 SAS assay with aggregated APP proteolytic fragments

When APP is cleaved by either the α- or β-secretase, larger fragments called sAPPα and sAPPβ are released extracellularly. We evaluated whether there was any cross-reactivity with sAPPα and SAPPβ fragments using the BAN50 SAS assay (Figure S3). Mixed between- and within-subject ANOVA analysis indicated that there was a significant three-way interaction of oligomerization status (monomer versus oligomer), sAPP fragment (alpha versus beta) and concentration (p = 0.0055, GG adjusted) due to a differential linear (p = 0.0052) change in signal across concentrations among subgroups. Post hoc tests showed a significant increase in signal for oligomeric sAPPα (F = 34.64, df = 5, 10, p = 0.03, GG adjusted). Cross-reactivity was not observed for monomeric sAPPα (F= 2.13, df = 5, 10, p = 0.28, GG adjusted), oligomeric sAPPβ (F = 0.69, df = 5, 10, p = 0.52, GG adjusted), or monomeric sAPPβ (F = 2.11, df = 5, 10, p = 0.25, GG adjusted). Therefore the cross-reactivity in the Ban50 assay that we observe is limited to oligomeric sAPPα, which to our knowledge has not been shown to exist at high levels in patient CSF.

In order to further examine the effect of free soluble sAPPα on the detection of aβ oligomers in the Ban50 assay *in vitro*, oligomeric aβ was kept constant while monomeric sAPPα was assayed at a range of physiologically relevant concentrations. One-way repeated measures ANOVA showed no significant relation of concentration to BAN50 signal overall (F = 0.34, df = 4, 8, p = 0.65, GG adjusted) or in its linear polynomial component (F = 0.31, df = 1,2, p = 0.63). We also performed immunodepletion using pooled CSFs from AD patients comparing signal after incubating with either sAPPα specific antibody 2B3 or control IgG and did not find a significant change in Ban50 reactivity (t(4) = 1.49, p = 0.21, independent measures t test). In human CSF, depleting sAPPα does not diminish the amount of BAN50 signal detected, supporting the conclusion that oligomeric sAPPα is not likely to be a major biological confound in patient samples.

The cross reactivity with sAPPα that we observe is consistent with prior studies indicating the Ban50 epitope is located at the N-terminus of aβ42 [4,39], which is present in sAPPα but not sAPPβ [40]. Cross-reactivity with sAPP fragments is worth evaluating within the context of the assay’s performance characteristics, however there is no consensus about whether sAPP proteolytic proteins increase [41], decrease [42] are unchanged [43–45] in AD and it is also not documented whether these proteins are aggregated under physiologic conditions. While the immunodepletion and *in vitro* experiments make it seem less likely that sAPPα oligomers account for the increased BAN50 SAS reactivity observed in AD CSF relative to normal controls, it is plausible that aβ42 or sAPPα species could influence the measurement of oligomeric species by interacting with capture or detect antibodies.

## Discussion

Previous studies investigating the presence of aβ oligomers in CSF from AD patients have employed a diverse range of techniques dedicated to investigating different species of aggregates. In designing this Luminex assay, we adapted a Ban50 single antibody sandwich ELISA that was previously shown to detect elevated HMW aβ oligomers in CSF [4]. Interestingly, this assay has previously been reported to detect a set of high molecular weight oligomers rather than dimer, trimer, tetramer or hexameric species of aβ due to the availability of the BAN50 epitope in oligomeric forms [4]. The initial report of the BAN50 single antibody sandwich ELISA technique found that measuring HMW oligomers of aβ could distinguish AD from control patients [4] and we were able to confirm these observations in the samples we utilized.

Another consideration in experimental design is whether the dynamic equilibrium of aβ monomers and oligomers are affected during the assay protocol and whether monomeric aβ42 present in CSF might affect oligomeric aβ measurements. Luminex assays are typically incubated overnight at room temperature on an orbital shaker to maximize interaction of capture antibodies coated on the microspheres with analytes and detect antibodies in solution, conditions that are especially important for CSF assays that detect low abundance proteins [46]. However incubating high concentrations of aβ42 under similar conditions has been shown to promote oligomerization and fibril formation *in vitro* [47]. It is unclear whether this is a significant confound because the *in vitro* studies were performed using superphysiologic concentrations of aβ42 in a matrix that is compositionally different from CSF.

Several other assay designs have been utilized to assay oligomeric aβ in human CSF. One of the most promising assays is a nanoparticle based approach that was shown to yield excellent discrimination between AD and normal controls. This ultrasensitive assay uses biobarcode amplification to enhance signal, with estimated concentration medians for amyloid-β-derived diffusible ligands at 200 aM and 1.7 fM [48], however this study was performed on an assay platform that has not yet been adapted for clinical use. Santos et al. have developed a unique assay that detects all species of oligomers by combining flow cytometry and fluorescence resonance energy transfer assay. Their group has found that aβ oligomers are detectable in CSF from non-demented control patients [17] as well as AD cases [15]. While the number of oligomers that they find are not significantly different in AD and control cases, they did observe an association between oligomers and MMSE scores [15]. Other groups using SAS design [16,18,19] or oligomer specific antibodies [19] did not find any difference in signal between AD and control CSF in their patient populations.

The variability in results from the many prior studies on oligomeric aβ in CSF may be due to many factors. Most of the published studies use a relatively small number of clinical samples on a variety of diagnostic platforms with different antibody pairs. Some assays have a greater avidity for LMW or HMW aβ species and therefore may report differently than assays that detect all oligomeric forms. While we were able to confirm the prior study showing increased levels of aβ oligomers in patient samples [4], we found that the oligomeric signal was very low relative to assays for tau, phosphorylated tau and aβ42. Moreover, while the ratio of oligomeric aβ to aβ42 reached a higher level of statistical significance in our samples compared to the standard aβ42 assay alone, the Alzbio3 system appears to distinguish control and AD patients as well as the current iteration of oligomeric aβ assays.

A major advance in the development of oligomeric aβ standards was recently reported by Kasai et al. who constructed a synthetic peptide connecting multiple BAN50 epitopes to a branching lysine core and was able to create a standard curve encompassing the physiological range of oligomeric aβ found in CSF [39]. Because versions of the oligomeric aβ assay both in this study and in prior publications [4] is read out in arbitrary units, using a branched chain synthetic oligomer standard should enable reliable quantification between assay runs. Based on our data, oligomeric aβ is a promising biomarker, however additional studies using well-standardized, high-sensitivity assays on large cohorts of patients will be needed before the analysis of oligomeric aβ becomes a diagnostically useful assay for the clinical evaluation of late onset AD patients.

## Supporting Information

Figure S1 Correlation with Clinical Dementia Rating (CDR), Delayed and Immediate Recall Scores. **A**. Scatterplot of CDR and aβ oligomers for the entire sample pooled with linear regression in red. The correlation is not statistically significant (Pearson correlation, r = 0.29, p = 0.07, n = 39, two-tailed) and is depicted with a solid red line. **B**. Scatterplot of Delayed Recall Score and aβ oligomers for the entire sample pooled with linear regression in red. The correlation is not statistically significant (Pearson correlation, r = -0.20, p = 0.22, n = 39, two-tailed). **C**. Scatterplot of Immediate Recall Score and aβ oligomers for the entire sample pooled with linear regression in red. The correlation is not statistically significant (Pearson correlation, r = -0.29, p = 0.07, n = 39, two-tailed).
(TIF)

Figure S2 The effect of storage time on assay outcomes. **A**. Scatterplot of aβ oligomers relative to sample storage time with linear regression in red. The correlation is not statistically significant (Pearson correlation, r = 0.114, p = 0.49, n=39, two-tailed). **B**. Scatterplot of aβ42 relative to sample storage time with linear regression in red. The correlation is not statistically significant (Pearson correlation, r = -0.143, p = 0.38, n=39, two-tailed) **C**. Scatterplot of tau relative to sample storage time with linear regression in red. The correlation is not statistically significant (Pearson correlation, r = -.00065, p = 1.00, n = 38, two tailed). **D**. Scatterplot of tau 181P relative to sample storage time with linear regression in red. The correlation is not statistically significant (Pearson correlation, r = 0.014, p = 0.93, n = 39, two-tailed).
(TIF)

Figure S3 Cross-reactivity of BAN50 SAS assay with monomeric and oligomeric sAPP fragments. **A**. Serial dilutions of monomeric or oligomeric sAPPα were assessed by BAN50 SAS assay. Mixed between- and within-subject ANOVA analysis followed by post hoc tests indicated that there was a significant increase in signal for oligomeric sAPPα (F = 34.64, df = 5, 10, p = 0.03, Greenhouse-Geisser (GG), adjusted). No significant relation between concentration and assay signal was noted for monomeric sAPPα (F= 2.13, df = 5, 10, p = 0.28, GG adjusted). Error bars represent standard deviations from three independent experiments. **B**. Serial dilutions of monomeric or oligomeric sAPPβ were assessed by BAN50 SAS assay. Mixed between- and within-subject ANOVA analysis followed by post hoc tests indicated that there was not a significant relationship between the assay signal and concentration of oligomeric sAPPβ (F = 0.69, df = 5, 10, p = 0.52, GG adjusted), or monomeric sAPPβ (F = 2.11, df = 5, 10, p = 0.25, GG adjusted). Error bars represent standard deviations from three independent experiments. **C**. Serial dilutions of monomeric sAPPα were added to samples containing constant levels of aβ oligomers were measured using the Ban50 assay to test whether soluble sAPP interferes with the measurement of oligomers *in vitro*. A one-way repeated measures ANOVA showed no significant relation of concentration to signal overall (F = 0.34, df = 4, 8, p = 0.65, GG adjusted). Error bars represent standard deviations from three independent experiments. **D**. Immunodepletion of human CSF with either sAPPα specific antibody 2B3 or control IgG was performed to test whether Ban50 reactivity in patient samples is due to sAPPα. NS indicates difference between sAPPα immunodepleted and control groups were not significantly significant (t(4) = 1.49, p = 0.21).
(TIF)
