# Supplementary figures and images for: A Luminex Assay Detects Amyloid β Oligomers in Alzheimer’s Disease Cerebrospinal Fluid
Source: PLoS One. 2013 Jul 2;8(7):e67898. doi: 10.1371/journal.pone.0067898 (PMC3699502; doi:10.1371/journal.pone.0067898)

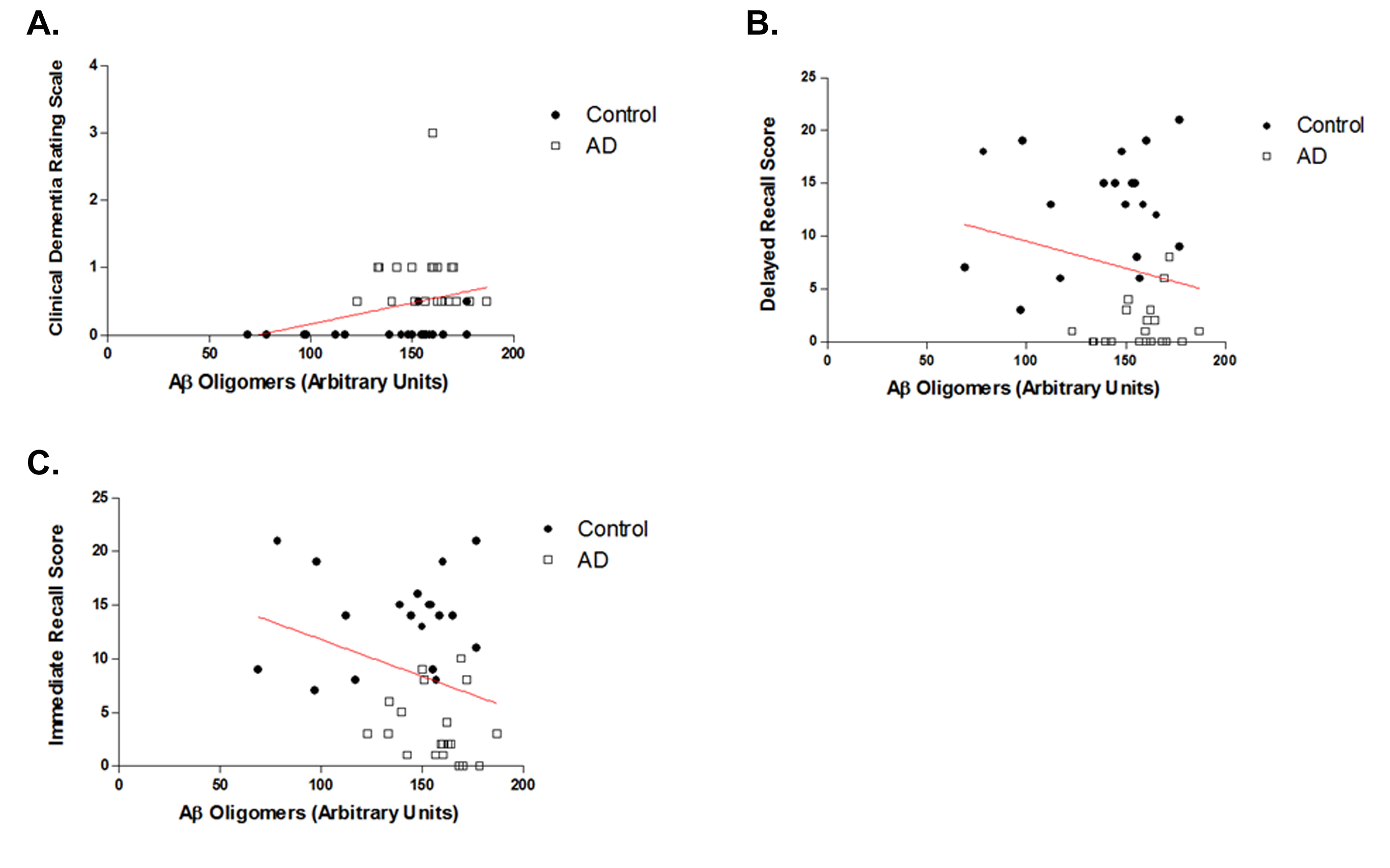

Supplement: Figure S1 — A. Scatterplot of CDR and aβ oligomers for the entire sample pooled with linear regression in red. The correlation is not statistically significant (Pearson correlation, r = 0.29, p = 0.07, n = 39, two-tailed) and is depicted with a solid red line. B. Scatterplot of Delayed Recall Score and aβ oligomers for the entire sample pooled with linear regression in red. The correlation is not statistically significant (Pearson correlation, r = -0.20, p = 0.22, n = 39, two-tailed). C. Scatterplot of Immediate Recall Score and aβ oligomers for the entire sample pooled with linear regression in red. The correlation is not statistically significant (Pearson correlation, r = -0.29, p = 0.07, n = 39, two-tailed). (TIF) [file pone.0067898.s001.tif]

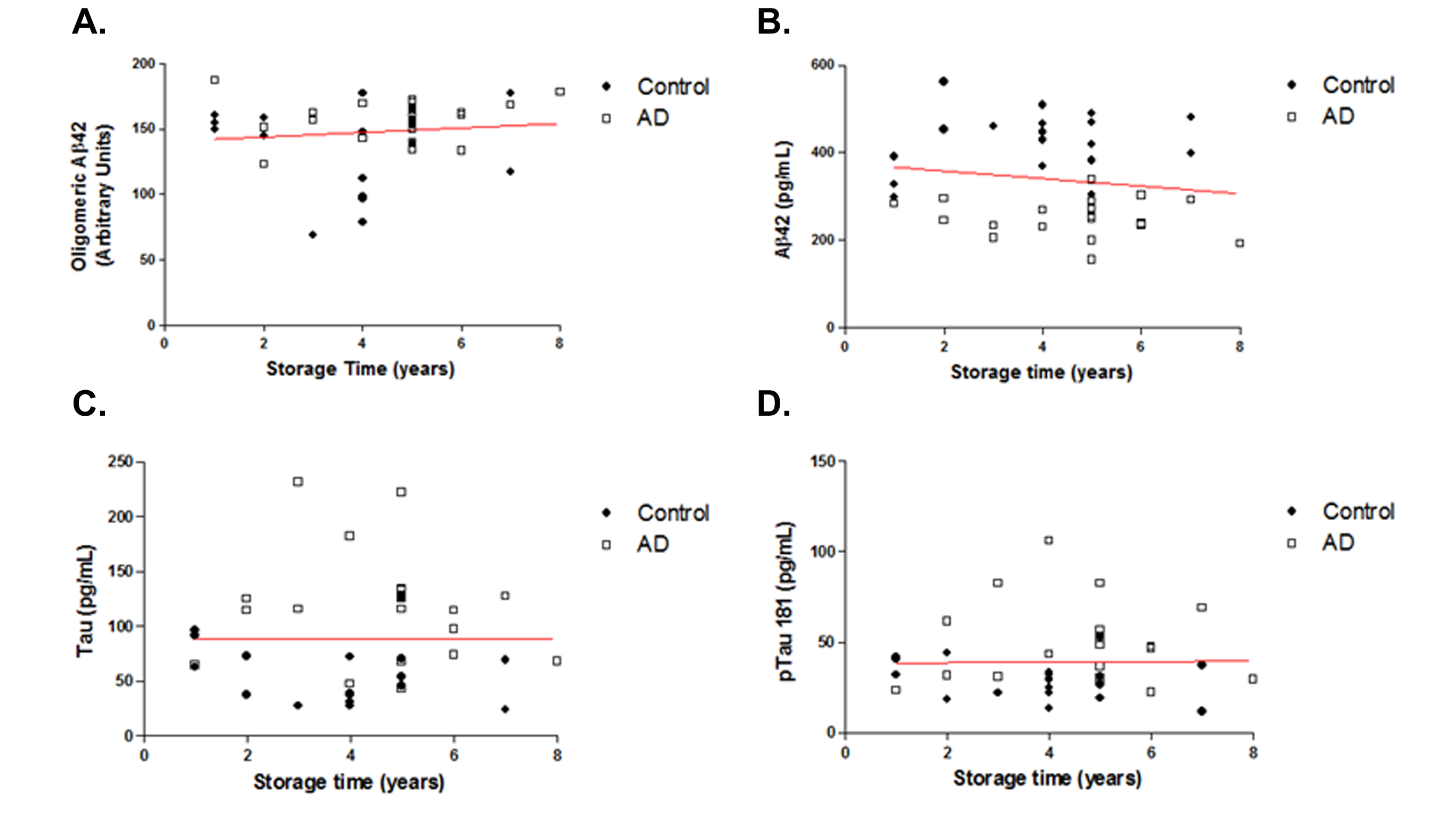

Supplement: Figure S2 — A. Scatterplot of aβ oligomers relative to sample storage time with linear regression in red. The correlation is not statistically significant (Pearson correlation, r = 0.114, p = 0.49, n=39, two-tailed). B. Scatterplot of aβ42 relative to sample storage time with linear regression in red. The correlation is not statistically significant (Pearson correlation, r = -0.143, p = 0.38, n=39, two-tailed) C. Scatterplot of tau relative to sample storage time with linear regression in red. The correlation is not statistically significant (Pearson correlation, r = -.00065, p = 1.00, n = 38, two tailed). D. Scatterplot of tau 181P relative to sample storage time with linear regression in red. The correlation is not statistically significant (Pearson correlation, r = 0.014, p = 0.93, n = 39, two-tailed). (TIF) [file pone.0067898.s002.tif]

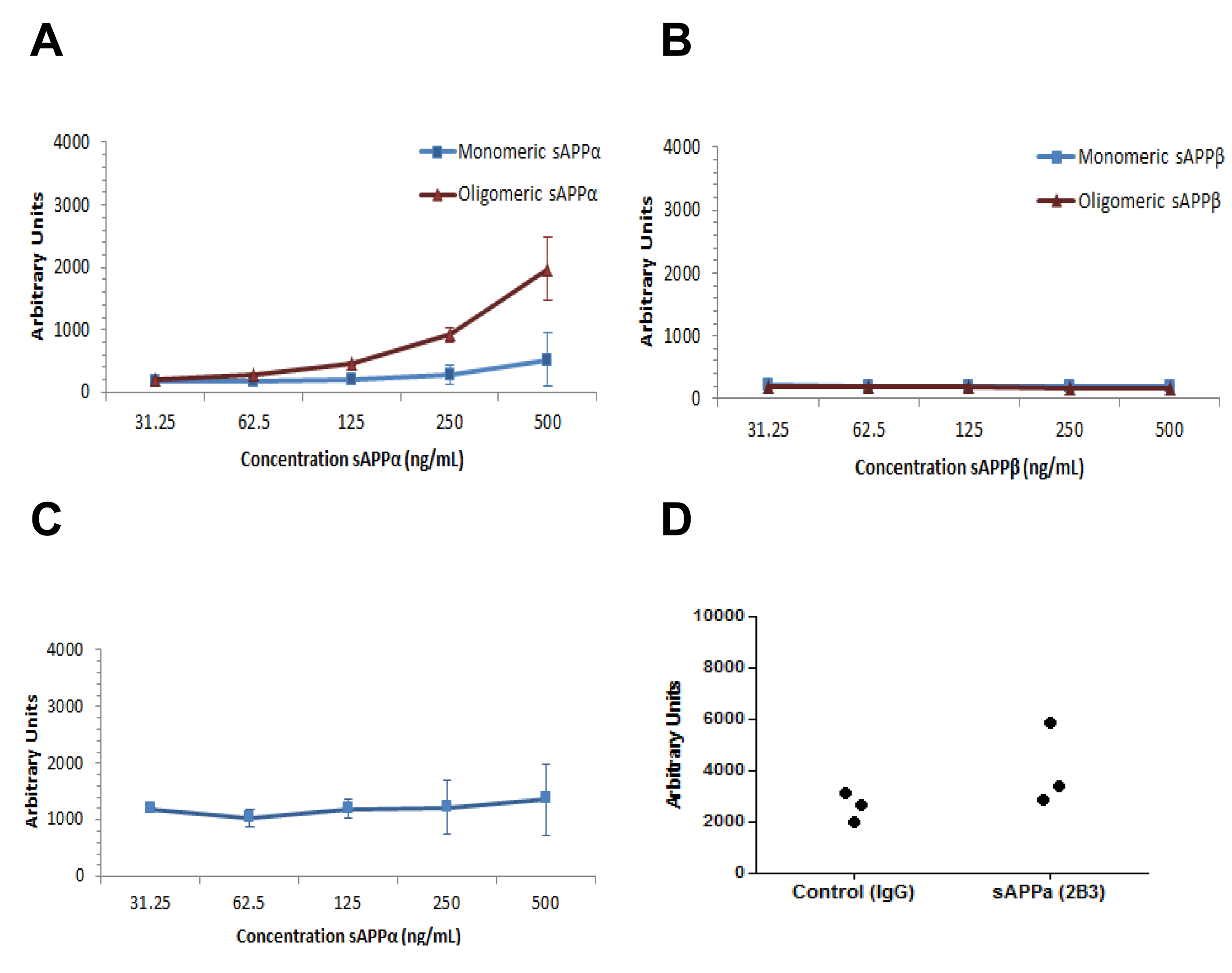

Supplement: Figure S3 — A. Serial dilutions of monomeric or oligomeric sAPPα were assessed by BAN50 SAS assay. Mixed between- and within-subject ANOVA analysis followed by post hoc tests indicated that there was a significant increase in signal for oligomeric sAPPα (F = 34.64, df = 5, 10, p = 0.03, Greenhouse-Geisser (GG), adjusted). No significant relation between concentration and assay signal was noted for monomeric sAPPα (F= 2.13, df = 5, 10, p = 0.28, GG adjusted). Error bars represent standard deviations from three independent experiments. B. Serial dilutions of monomeric or oligomeric sAPPβ were assessed by BAN50 SAS assay. Mixed between- and within-subject ANOVA analysis followed by post hoc tests indicated that there was not a significant relationship between the assay signal and concentration of oligomeric sAPPβ (F = 0.69, df = 5, 10, p = 0.52, GG adjusted), or monomeric sAPPβ (F = 2.11, df = 5, 10, p = 0.25, GG adjusted). Error bars represent standard deviations from three independent experiments. C. Serial dilutions of monomeric sAPPα were added to samples containing constant levels of aβ oligomers were measured using the Ban50 assay to test whether soluble sAPP interferes with the measurement of oligomers in vitro. A one-way repeated measures ANOVA showed no significant relation of concentration to signal overall (F = 0.34, df = 4, 8, p = 0.65, GG adjusted). Error bars represent standard deviations from three independent experiments. D. Immunodepletion of human CSF with either sAPPα specific antibody 2B3 or control IgG was performed to test whether Ban50 reactivity in patient samples is due to sAPPα. NS indicates difference between sAPPα immunodepleted and control groups were not significantly significant (t(4) = 1.49, p = 0.21). (TIF) [file pone.0067898.s003.tif]
